# Synthesis and Characterization of Cardanol-Based Non-Isocyanate Polyurethane

**DOI:** 10.3390/polym15244683

**Published:** 2023-12-12

**Authors:** Yanan Li, Bin Zhang, Yuzhuo Zhao, Shuai Lu, Donglei Fan, Song Wang, Jie Liu, Tao Tang, Sanxi Li

**Affiliations:** 1 School of Environmental and Chemical Engineering, Shenyang University of Technology, Shenyang 110870, China; lyn@smail.sut.edu.cn (Y.L.); wangsong@sut.edu.cn (S.W.); 2 State Key Laboratory of Polymer Physics and Chemistry, Changchun Institute of Applied Chemistry, Chinese Academy of Sciences, Changchun 130022, China

**Keywords:** non-isocyanate polyurethane, cyclic carbonate, cardanol, epoxidation

## Abstract

This paper describes the synthesis of NIPU by using cardanol as starting material. A cardanol formaldehyde oligomer was first prepared through the reaction of cardanol and formaldehyde, catalyzed by citric acid. The resulting oligomer was then subjected to epoxidation with m-chloroperbenzoic acid to obtain an epoxide compound, which was subsequently used to fix carbon dioxide (CO_2_) and form a cyclic carbonate. Using this cyclic carbonate, along with an amine, cardanol-based isocyanate polyurethane (NIPU) was prepared. Different characterization methods, such as Fourier transform infrared spectroscopy (FTIR), proton nuclear magnetic resonance (NMR), gel permeation chromatography (GPC), and thermogravimetric analysis (TGA), were used to confirm the synthesis of the four intermediate products and NIPU in the reaction process. This study highlights the promise of bio-based NIPU as a sustainable alternative in a number of applications while offering insightful information on the synthesis and characterization of the material.

## 1. Introduction

It is well known that the traditional polyurethane (PU) is usually synthesized from toxic isocyanate and polyol. Therefore, due to concerns about energy and health issues, strategies for developing green and sustainable biomass resources instead of isocyanate to prepare non-isocyanate polyurethane have important scientific significance and application value [1,2,3]. Non-isocyanate polyurethane made from renewable resources is referred to as bio-based polyurethane. Bio-based polyurethane is created utilizing renewable ingredients sourced from plants, animals, or microbes, as opposed to conventional polyurethane production procedures, which call for the use of hazardous substances like isocyanate [4,5]. As a result, there will be less of an adverse environmental impact and less reliance on conventional petrochemical raw materials. Textiles, coatings, packaging materials, and other industries frequently employ bio-based polyurethane [6,7,8,9,10]. However, bio-based polyurethane research is still in its early stages. In the age of green chemistry and an increased focus on process sustainability, numerous investigations on bio-based polyurethane have been carried out. Due to their unique properties of chemical and mechanical resistance, polymeric materials are widely used in all kinds of products in our daily life [11,12,13,14]. Using a non-isocyanate method, Ivan Javni et al. [15] produced polyurethane by reacting soybean oil carbonate with various diamines. They also looked at how the ratio of carbonate to amine and the amine structure affected the structural, mechanical, physical, and swelling characteristics of the polyurethane. With the use of epoxidation, hydroxylation, and curing processes, Jiali Liu et al. [16] effectively created a bio-based flexible polyurethane from waste palm oil and used it in urea-based coatings. The popularization of PU applications in medicine was limited by the toxicity of 4,4-methylene diphenyl diisocyanate (MDI). Consequently, PU made with MDI has found applications in rigid and flexible foams, coatings, adhesives, sealants, and elastomers. The variety of products, which determines the wide range of applications, is due to the variability of PU formulations [17]. From bio-based 1,3-propanediol and various chain length fatty diacids, Mengmeng Ruan et al. [18] created a variety of innovative bio-based aliphatic polyols that can be combined with 4,4′-methylene diphenyl diisocyanate to create reactive polyurethane hot melt adhesives. Henri Vahabi et al. [19] researched and clarified the chemical structure of bio-based flame-retardant polyurethane from the standpoint of flame retardancy as part of their study on the creation of flame-retardant bio-based polyurethane. To address the needs of practical applications, more advancements in synthesis techniques and performance are required.

As a naturally occurring phenolic compound obtained from biomass, cardanol can substitute phenol made from petroleum and minimize its detrimental effects on the environment [20]. In recent years, cardanol has been used in a variety of composite materials, including phenolic resins, epoxy resins, rubber, polyurethane, and acrylic, and it is often utilized in the pharmaceutical and cosmetics sectors since it demonstrates biological activities including antioxidant, antibacterial, and anti-inflammatory characteristics [21,22,23,24]. This study investigates the synthesis of bio-based non-isocyanate polyurethane using cardanol as a raw material, which is of great significance for the development of sustainable and environmentally friendly materials. This research on bio-based polyurethane aims to develop sustainable alternatives to reduce reliance on limited resources and minimize environmental burdens [25].

Moreover, in recent years, CO_2_ has been regarded as nontoxic, nonflammable, renewable, inexpensive, and an abundant C1 feedstock for organic synthesis. From the standpoint of environmental protection and resource utilization, chemical fixation of CO_2_ to obtain value-added organic chemicals under mild reaction conditions is an attractive approach. Many efficient routes for chemical CO_2_ fixation have been developed [26]. With 100% atom-economical conversion, the cycloaddition of epoxides to CO_2_, affording five-membered cyclic carbonates, is one of the most promising pathways [27].

To better understand the synthesis of cardanol-based nonisocyanate, the effects of different molar ratios of cardanol to formaldehyde and experimental reaction temperatures on the condensation reaction are compared to speculate the influence of the reaction conditions on the synthesis of cardanol formaldehyde aldehyde oligomer. After determining the optimal reaction conditions, the modification conditions for epoxidation, esterification, and amine curing of phenol-aldehyde oligomer are explored to synthesize non-isocyanate polyurethane using a route with fewer harmful substances. The cardanol formaldehyde oligomer (CFO) has been synthesized through the condensation of cardanol with formaldehyde (ratio of 1:0.7) in the presence of a citric acid catalyst. 3-chloroperbenzoic acid (0.20 mol) was added slowly into the flask with CFO (0.10 mol), and the reaction was carried out at 0 °C in an ice bath for 3 h. For the reaction between epoxides and carbon dioxide using TBAB as a catalyst, CO_2_ was added to the reactor until the pressure in the reactor reached 1.5 MPa. The reactants were stirred at 85 °C for 15 h. We are proposing a synthetic route to produce biobased NIPU from a cyclic carbonate and 1,6-hexanediamine. After the reactions, the NIPU was obtained as brown brittle solids. The experimental results indicate that a four-step reaction route from bio-based cardanol to non-isocyanate polyurethane has been established, providing a beneficial pathway for the high-value utilization of cardanol as a biomass raw material and the research and development of novel bio-based non-isocyanate polyurethane. Exploring the use of more environmentally friendly and renewable resources to further replace harmful substances in auxiliary materials is a future experimental arrangement.

## 2. Methodology

### 2.1. Chemicals and Reagents

Cardanol was supplied by Jining Huakai Resin Co., Ltd. (Jining, China). Aqueous formaldehyde (37%) was obtained from Liaoning Jiacheng Fine Chemicals Co., Ltd. (Fuxin, China). Methanol was purchased from Tianjin Fuyu Fine Chemical Co., Ltd. (Tianjin, China). 3-chloroperbenzoic acid (85%) was purchased from Shanghai Macklin Biochemical Technology Co., Ltd. (Shanghai, China). CO_2_ (99.99%) was supplied by Shenyang Runfeng Specialty Gas Co., Ltd. (Shenyang, China). Citric acid (99.5%) and hexanediamine were bought from Tianjin Damao Chemical Reagent Factory (Tianjin, China).

### 2.2. Experimental Equipment

A magnetic stirrer (DW–1–60), circulating vacuum pump (DF–101S), and high-temperature oil bath pot (SHZ–D (Ⅲ)) were obtained from Gongyi Yuhua instrument Co., Ltd. (Zhengzhou, China). A low temperature thermostat (DC–0515) was purchased from Jiangsu Tianling Instrument Co., Ltd. (Yancheng, China). The flange magneton reactor was supplied by Shanghai Huotong Experimental Instrument Co., Ltd. (Shanghai, China). The electric blast drying oven was supplied by Shanghai Boxun Industrial Co., Ltd. (Shanghai, China).

### 2.3. Experimental Method

#### 2.3.1. Synthesis and Purification of Cardanol Formaldehyde Oligomer

**Scheme 1:**
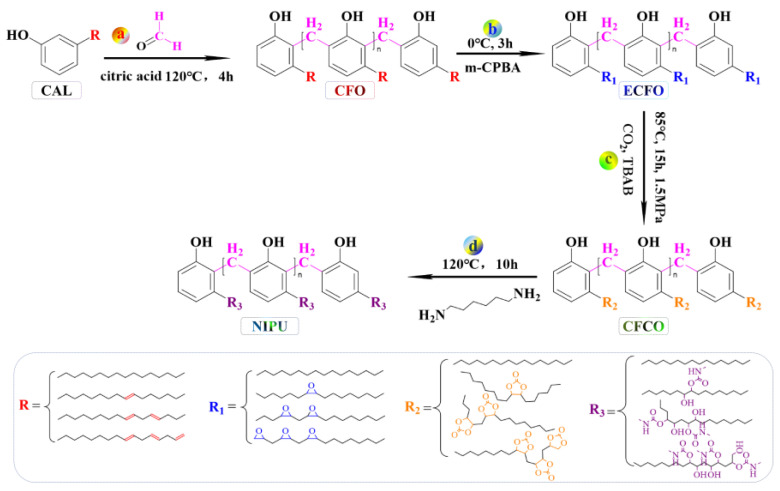
Synthesis of cardanol formaldehyde oligomer (CFO (**a**)), epoxidized cardanol formaldehyde oligomer (ECFO (**b**)), cardanol formaldehyde cyclocarbonate oligomer (CFCO (**c**)), and cardanol-based non-isocyanate polyurethane (NIPU (**d**)).

**Scheme 2:**
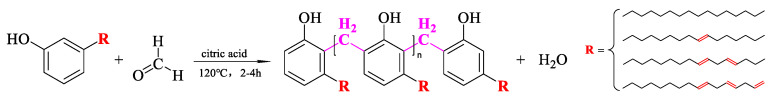
Synthesis of cardanol formaldehyde oligomer (CFO).

**Scheme 3:**
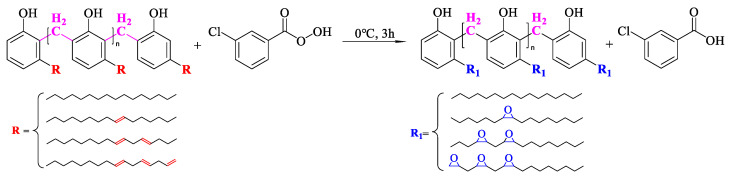
Synthesis of epoxidized cardanol formaldehyde oligomer (ECFO).

**Scheme 4:**
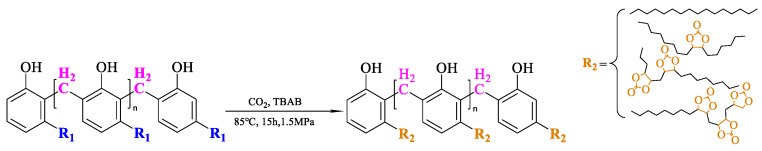
Synthesis of cardanol formaldehyde cyclocarbonate oligomer (CFCO).

**Scheme 5:**
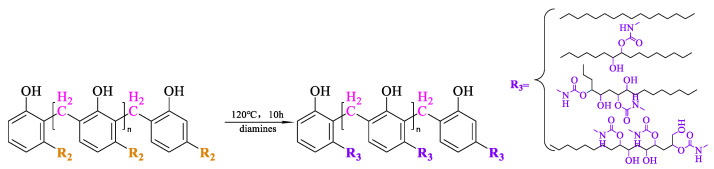
Synthesis of cardanol-based non-isocyanate polyurethane (NIPU).

A general overview of the experiment is shown in Scheme 1. The synthesis of cardanol formaldehyde oligomer (CFO) is demonstrated in Scheme 1a. The synthesis and purification of epoxidized cardanol formaldehyde oligomer (ECFO) is shown in Scheme 1b. The synthesis of cardanol formaldehyde cyclocarbonate oligomer (CFCO) and non-isocyanate polyurethane (NIPU) are illustrated in Scheme 1c,d. The specific reaction equations of every step are shown in the experimental Scheme 2, Scheme 3, Scheme 4 and Scheme 5.

This scheme is split as follows:

The chemical equation for the reaction is demonstrated in Scheme 1a and Scheme 2. The cardanol liquid was accurately weighed and transferred into a four-necked flask. In a beaker, 1 wt% citric acid was dissolved in 2 mL of methanol through gently shaking until it was fully dissolved. At room temperature, the mixture of formaldehyde and citric acid was slowly added to a four-necked flask containing the cardanol. After complete addition, the mixture was stirred at room temperature, the temperature of the oil bath was gradually increased to 120 °C, and the reaction mixture was refluxed for 4–5 h [28]. Once the reaction was complete, the system was cooled and an appropriate amount of ethyl acetate was carefully added, followed by the addition of a suitable amount of distilled water at 60 °C while stirring, for washing. The resulting mixture was transferred to a separatory funnel and allowed to stand for phase separation, ensuring that the organic layer was retained. The ethyl acetate and water were removed from the organic layer using reduced pressure using distillation techniques. Finally, the desired product, cardanol formaldehyde oligomer (CFO), was collected.

#### 2.3.2. Synthesis and Purification of Epoxidized Cardanol Formaldehyde Oligomer (ECFO)

The cardanol formaldehyde oligomer was poured into a four-orifice round-bottom flask equipped with a magnetic agitator, and an appropriate amount of mixed drops of 3-chloroperoxybenzoic acid crystals and dichloromethane were added to the flask. An ice bath reaction was then carried out at 0 °C for 3 h [29]. After the reaction was ceased, the filtrate was pumped and retained, and then the filtrate was removed from the dichloromethane solvent to obtain a solid–liquid mixture. Then, ethyl acetate was added to dissolve the solid–liquid mixture, and then 5% solidople was used to wash away the possible residual m-chloroperoxybenzoic acid and the by-product m-chlorobenzoic acid. The blending system was washed with distilled water and was then poured into a separation funnel to stand the separation liquid, leaving the organic layer. Then, the ethyl acetate and water were removed, leaving the organic epoxidized cardanol formaldehyde oligomer (ECFO). The chemical equation for the reaction is shown in Scheme 1b and Scheme 3.

#### 2.3.3. Synthesis of Cardanol Formaldehyde Cyclocarbonate Oligomer (CFCO)

An epoxidized cardanol formaldehyde oligomer, tetrabutylammonium bromide, was added to a teflon reaction vessel in an autoclave. After the autoclave was secured, CO_2_ gas was transferred into the autoclave to purge it twice, then the CO_2_ was passed to increase the tension to 2.0 MPa. When the pressure was stable, the reaction conditions of the autoclave were set to 85 °C, 300 r/min, and 900 min. A brown–reddish-brown viscous fluid cardanol formaldehyde cyclocarbonate oligomer was obtained, which was more viscous than the epoxidized cardanol formaldehyde oligomer. The chemical equation for the reaction is depicted in Scheme 1c and Scheme 4.

#### 2.3.4. Synthesis of Cardanol-Based Non-Isocyanate Polyurethane

The chemical equation for the reaction is illustrated in Scheme 1d and Scheme 5. The viscosity of the synthesized cardanol formaldehyde cyclocarbonate oligomer was very high; therefore, before each sampling, the required cardanol formaldehyde cyclocarbonate oligomer was heated in the oven and sampled into a single-mouth bottle. The curing agent hexenediamine was added to the single-mouth bottle and mechanically stirred for 12 h to make the two components fully and evenly mixed, and the mixed raw materials were injected into the preheated mold. The mold was placed in the vacuum oven, and the vacuum pressure was approximately −0.1 MPa. Then, the temperature was raised to 140 °C for 30 h so that the spline could avoid bubbles during pre-curing. The sample was taken out of the mold, placed into the atmospheric pressure drying oven for 10 h, and the spline was formed. The mold was taken out and cooled naturally to obtain NIPU-cured spline.

#### 2.3.5. Characterization of the Prepared Samples: Analysis Method

The Fourier transform infrared spectra FTIR were measured using a IR Prestige 21 from Shimadzu Company (Tokyo, Japan) to monitor the various functional groups within the wavelength range of 400 cm^−1^~4000 cm^−1^. Nuclear magnetic resonance (NMR) analysis was carried out using a Bruker AVANCEIII 400 MHz NMR spectrometer (Ettlingen, Germany), using deuterated chloroform as solvent. Gel permeation chromatography (GPC) was performed using an apparatus consisting of a PolymerLabs PL-GPC 50 Plus (California, America), and THF was used as the mobile phase and the stationary phase in the experiment. To study the curing kinetics of the samples, a thermogravimetric analysis (TGA) was conducted using a STA449 C/41 G thermal analyzer (America) under both air and nitrogen atmospheres, with heating conditions increasing from 25 to 900 °C under a ramp rate of 20 °C/min.

## 3. Results and Discussion

### 3.1. Synthesis and Characterization of Cardanol Formaldehyde Oligomer (CFO)

**Figure 1:**
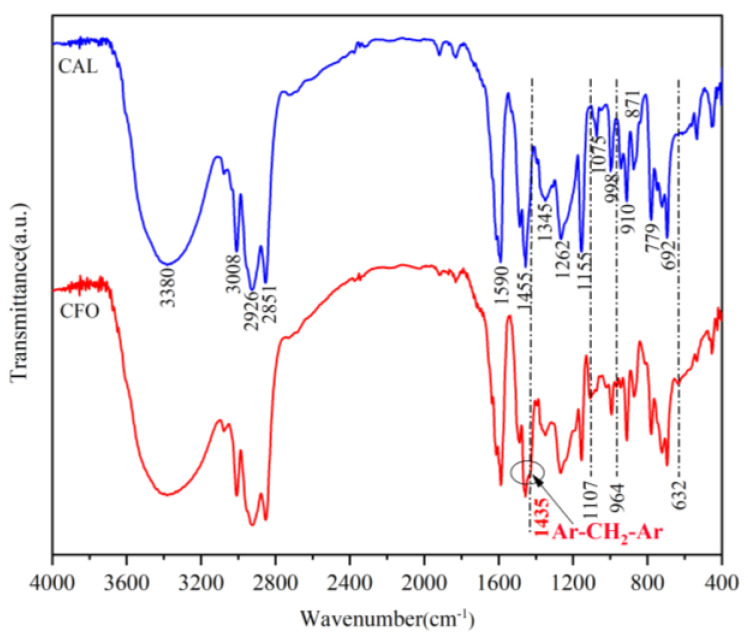
FTIR spectra of cardanol (CAL) and cardanol formaldehyde oligomer (CFO).

The methylation of cardanol is catalyzed by the reaction of citric acid with formaldehyde. The release of hydrogen ions from the reaction between cardanol and o-hydroxymethyl causes the acidity of the reaction mixture to increase, thereby reducing the pH of the reaction mixture [30]. The FTIR spectral analysis of the cardanol formaldehyde oligomer synthesized from cardanol with formaldehyde and raw cardanol not only revealed the condensation of methylated cashew phenol, but also revealed the degree of ortho-substitution and para-substitution [31]. As shown in Figure 1, the wide peak centered at 3380 cm^−1^ in the infrared spectrum of cardanol can be attributed to the vibration of the O-H bond of the phenol group, as well as the characteristic peak of the in-plane bending vibration at 1345 cm^−1^. The stretching vibrations of unsaturated double bonds in cardanol alkyl carbon chains and the antisymmetric and symmetric stretching vibrations of methyl and methylene are 3008, 2926 and 2851 cm^−1^, respectively. The absorption peaks of C=C and C-H stretching vibration on the carbon skeleton of benzene ring are 1590 and 1455 cm^−1^, respectively. The tensile vibration absorption peaks of C-O between the phenol hydroxyl group and the benzene ring group can be seen at 1262 and 1155 cm^−1^ [6,29]. The small peak near 910 cm^−1^ may be due to the presence of three adjacent hydrogen atoms in the nucleus of benzene. The peak found at 1267 cm^−1^ is associated with the presence of C-O extended aromatic rings. The double bond corresponding to the side chain (-C=C-) was observed at 871–998 cm^−1^, 692, and 779 cm^−1^ [32]. The spectra of CFO, 3008 cm^−1^ (C-H tensile vibration of olefin) and 720 cm^−1^ (C-H out-of-plane deformation), were almost unaffected, suggesting that polymerization occurs through the substitution of CH_2_OH rather than through double bonds in the side chain. Further observation of the difference between the newly synthesized cardanol formaldehyde oligomer (CFO) and cardanol (CAL) showed that the stretching vibration absorption peaks, including -OH, shifted to lower wave numers. The peaks near 964 and 998 cm^−1^ were the substitutions in benzene nuclei, and the peaks at 964 and 1107 cm^−1^ were due to the ortho-substitutions in benzene nuclei. A new peak at approximately 1435 cm^−1^ indicates the presence of methylene (Ar-CH_2_-Ar), which connects the two benzene rings. The emergence of new FTIR absorption peaks confirmed the polycondensation reaction between cardanol and formaldehyde to form a cardanol formaldehyde oligomer [33].

**Figure 2:**
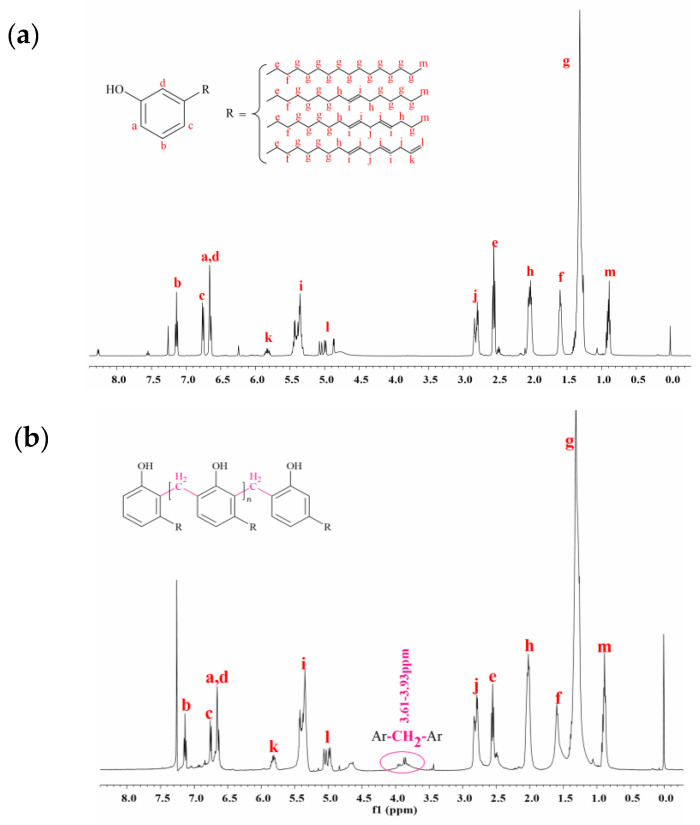
^1^H NMR spectra of cardanol (CAL, (**a**)) and cardanol formaldehyde oligomer (CFO, (**b**)).

Figure 2a shows the ^1^H NMR spectrum of cardanol, and the peak values at δ 6.6–7.14 ppm are attributed to aromatic matrix elements (H-b, H-c and H-a, d) in the benzene ring, respectively [34]. δ 4.98–5.83 ppm is the ethylene olefins proton (C=CH_2_) of the long aliphatic side chain, and the peaks at δ 0.89–2.80 ppm are the methylene and the terminal methyl groups of the long aliphatic side chain, respectively. The strong peaks near δ 1.31 ppm belong to long aliphatic side chains of methylene (more than five). In Figure 2b, the peak value of δ 6.66–7.14 ppm in the cardanol formaldehyde oligomer (CFO) spectrum is due to the aromatic matrix of benzene nucleus, and the peak position is virtually unchanged from that of raw cardanol. Notably, new peaks near δ 3.61–3.93 ppm indicate methylene protons (Ar-CH_2_-Ar) at the bridge between the benzene rings [34,35].

**Figure 3:**
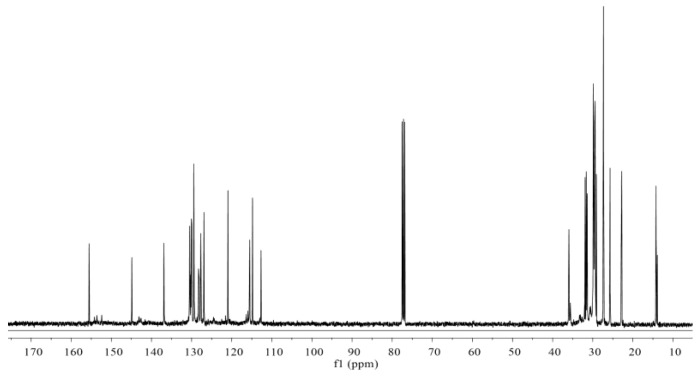
^13^C-NMR spectrum of cardanol formaldehyde oligomer (CFO).

In the ^13^C-NMR spectrum of cardanol formaldehyde oligomer (Figure 3), the resonance associated with the bridging methylene carbons are characteristic of novolac resins. The appearance of a signal at 30.5 ppm clearly evidences the presence of an ortho–ortho bridge carbon, which indicates that one cardanol molecule is attached to another cardanol molecule through the methylene group. The peak at 156 ppm corresponds to the phenolic –OH attached to carbon, and the substituted meta carbon of cardanol resonated between 128 and 136 ppm. The peaks at 17–36 ppm are due to the methylene carbons of long aliphatic side chains of cardanol [32].

The synthetic cardanol formaldehyde oligomer was tested using gel permeation chromatography, and the test results are shown in Table 1. In Table 1, peaks 1 and 2 are the results of the molecular weight and polydispersity index of the cardanol formaldehyde oligomer and raw cardanol, respectively. It can be seen from the table that the average molecular weight of cardanol is 354 g/mol and that of the cardanol formaldehyde oligomer is 5131 g/mol, indicating that the synthesized cardanol formaldehyde oligomer is an oligomer of cardanol monomer, and the polymerization degree *n* is approximately 14. This shows that the reaction had a good effect in the process of synthesizing cardanol formaldehyde oligomer.

### 3.2. Synthesis and Characterization of Epoxidized Cardanol Formaldehyde Oligomer (ECFO)

**Figure 4:**
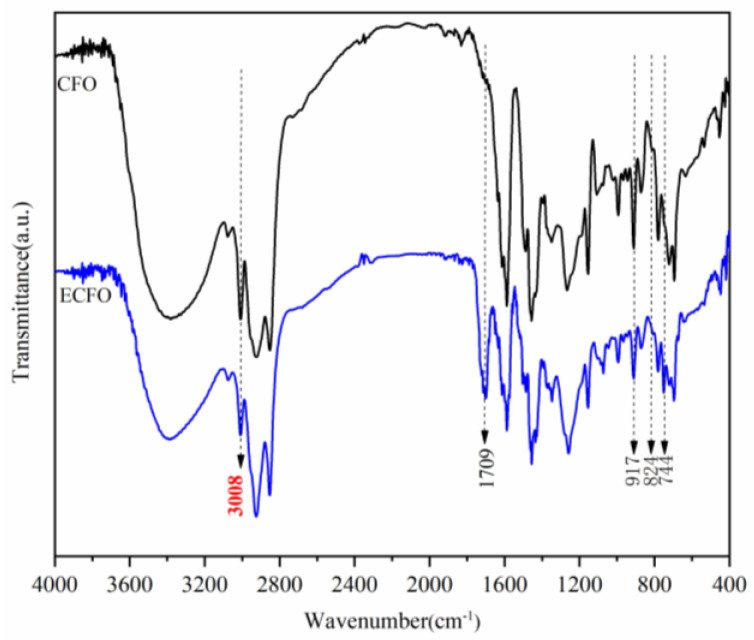
FTIR spectra of cardanol formaldehyde oligomer (CFO) and epoxidized cardanol formaldehyde oligomer (ECFO).

The infrared spectra of epoxidized cardanol formaldehyde oligomer (ECFO) and cardanol formaldehyde oligomer (CFO) are shown in Figure 4. First, the peak of C-H in the inner unsaturated moiety at 3008 cm^−1^ is absent due to the conversion to epoxide. Second, the characteristic features of the epoxy group are found at 744, 824, and 917 cm^−1^. Furthermore, the typical peak of the phenolic hydroxyl group still exists. These indicate that the cardanol was converted into epoxidized cardanol formaldehyde oligomer [36]. This indicates the occurrence of an etherification reaction, where some double bonds were epoxidized, resulting in the formation of epoxy alkyl groups [30,35]. These peaks almost confirm the structure of the epoxidized cardanol formaldehyde oligomer. It should be noted that some epoxy absorption peaks often overlap with absorption peaks of other functional groups around 820–920 cm^−1^. Therefore, when interpreting the infrared spectrum, a comprehensive analysis and identification must be performed by combining other characteristic vibrations [23]. The strong peak observed at 1709 cm^−1^ in the reaction product corresponds to the stretching vibration absorption peak of the carbonyl group (C=O). For the occurrence of this special peak, we have given many speculations, which may be partly due to the acidic conditions in the previous reaction leading to the subsequent epoxy ring opening reaction and the formation of carbonyl compounds. It may also be partially due to the esterification of acids (-COOH) and phenols (-OH).

**Figure 5:**
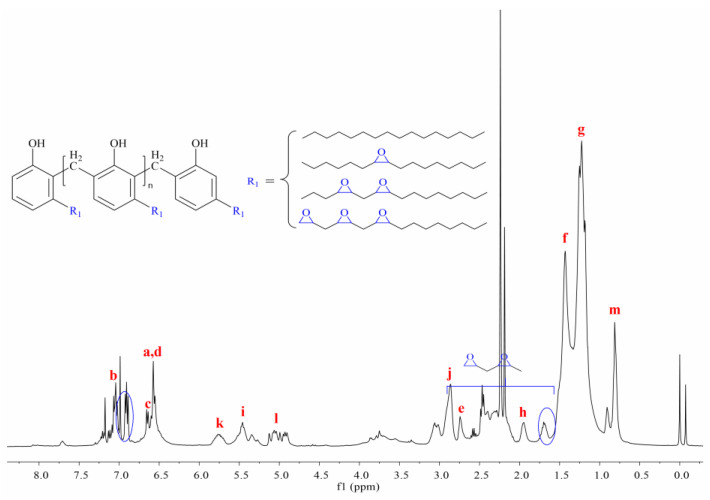
^1^H NMR spectra of epoxidized cardanol formaldehyde oligomer (ECFO).

In the ^1^H NMR spectrum of epoxidized cardanol formaldehyde oligomer (ECFO), as shown in Figure 5, the positions of most peaks are not shifted compared to CFO. After epoxidation, the signal corresponding to the olefinic proton on the side chain almost disappears in the ^1^H NMR spectrum of ECFO. Additionally, the new peaks at 2.88–3.02 ppm indicate epoxy groups in ECFO [24,29]. Furthermore, the changed chemical shift of the peaks at 1.3–1.8 ppm also supports the formation of epoxidized groups [36]. The information in the infrared spectrum further confirms that ECFO containing an epoxy structure was successfully synthesized in the reaction.

**Figure 6:**
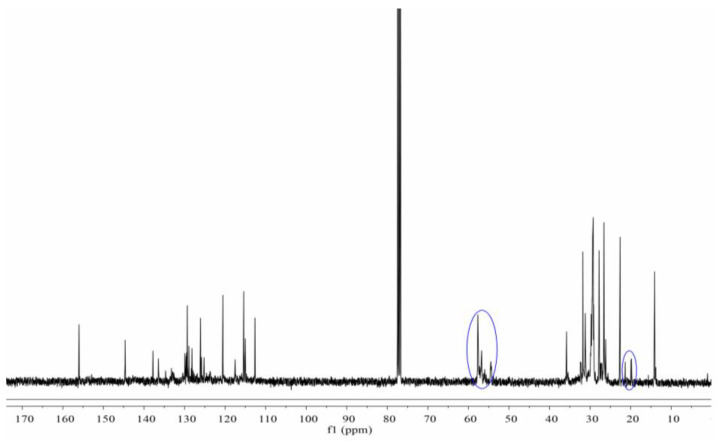
^13^C-NMR spectrum of epoxidized cardanol formaldehyde oligomer (ECFO).

In the case of ^13^C-NMR, signals corresponding to the methoxy and –CH carbon were observed at 54.5 and 58 ppm. The resonance peaks at 21 and 22 ppm may be assigned to –CH_2_ carbons of the oxirane ring. The remaining peaks were similar to the peaks present in the backbone structure of the novolac resin, as shown in Figure 6 [32,36].

### 3.3. Synthesis and Characterization of Cardanol Formaldehyde Cyclocarbonate Oligomer (CFCO)

**Figure 7:**
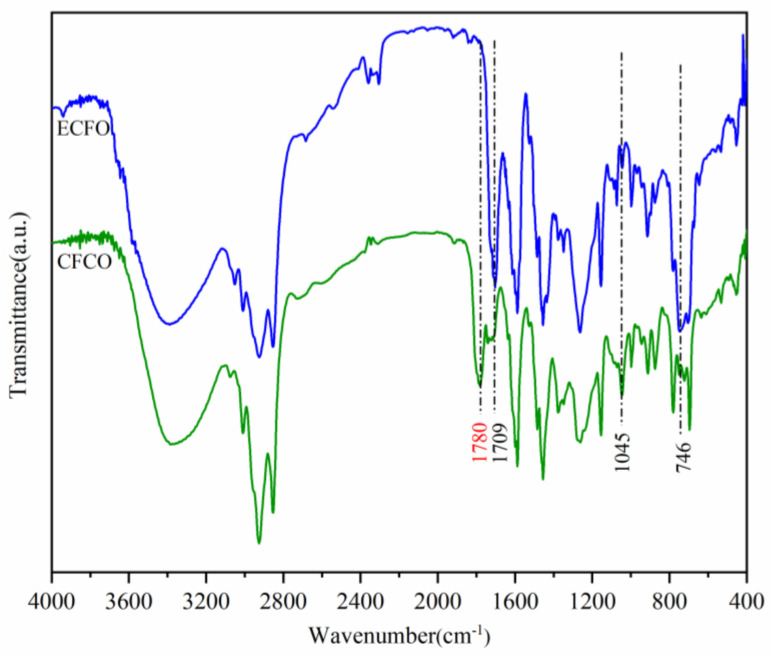
FTIR spectra of epoxidized cardanol formaldehyde oligomer (ECFO) and cardanol formaldehyde cyclocarbonate oligomer (CFCO).

The infrared spectrum in Figure 7 depicts the synthesized cardanol formaldehyde cyclocarbonate oligomer (CFCO) and the epoxidized cardanol formaldehyde oligomer (ECFO). As shown in the FT-IR spectrum of the cyclic carbonate, the characteristic absorption peak of the epoxide groups disappeared, and two new characteristic absorption peaks, which were ascribed to the C=O and C–O bonds on the five-membered cyclic carbonate rings, appeared at 1780 and 1045 cm^−1^, respectively [1,37]. This indicates that the epoxy groups reacted with CO_2_, converting them into a five-membered cyclic carbonate structure.

**Figure 8:**
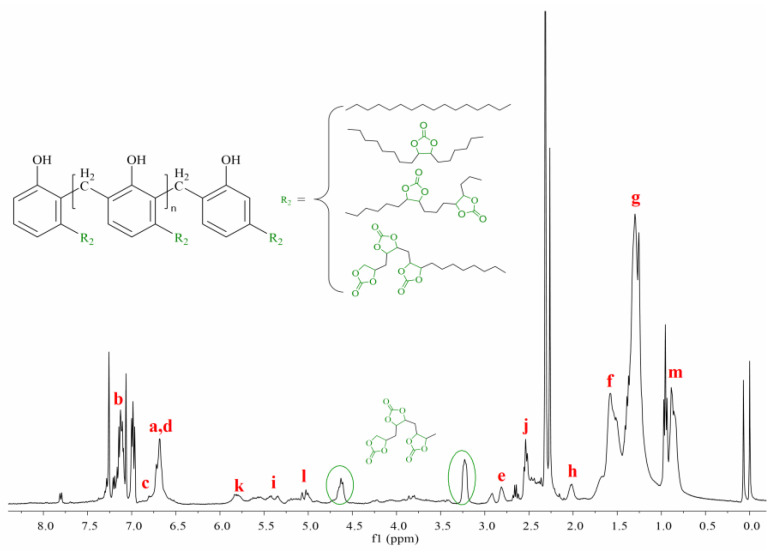
^1^H NMR spectra of cardanol formaldehyde cyclocarbonate oligomer (CFCO).

Figure 8 displays the proton nuclear magnetic resonance (^1^H NMR) spectrum of cardanol formaldehyde cyclocarbonate oligomer (CFCO), further confirming its chemical structure. After the insertion of CO_2_ into the epoxide rings, the signal of the methine protons originally located on the epoxide rings was shifted from 2.88–3.02 ppm to 5.14–5.19 ppm, which is the characteristic signal region of the methine protons of the cyclic carbonate rings [26]. The signal at 4.60–4.65 ppm was assigned to the methylene protons adjacent to the cyclic carbonate groups. The signal at 0.85 and 3.21 ppm may be assigned to the five-membered cyclic carbonate rings, which were ascribed to the C=O and C–O bonds. The ^1^H NMR results further validated the cycloaddition of oxirane groups with CO_2_ for the formation of cyclic carbonate groups. No epoxy compounds were detected, indicating that the cyclic carbonate product obtained was of a high purity [37]. This peak can be attributed to the methyl protons on the newly formed cyclic carbonate group. The signal corresponding to the protons on the CFCO ring did not shift substantially due to the similar chemical environment.

**Figure 9:**
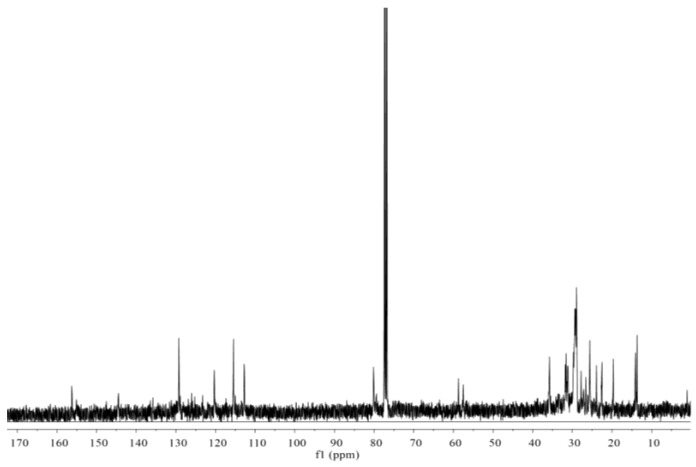
^13^C-NMR spectrum of cardanol formaldehyde cyclocarbonate oligomer (CFCO).

The TBAB catalyst was previously employed as a green and efficient catalyst for the cycloaddition of CO_2_ with epoxides. Therefore, it was also adopted for the conversion of a complete epoxy group consumption during CO_2_ cycloaddition, as no residual epoxide peaks remained in the ^13^C NMR spectra of the synthesized cardanol formaldehyde cyclocarbonate oligomer samples which is shown in Figure 9. Moreover, the characteristic carbon signals of the cyclic carbonate ring at 155.8 ppm (C=O), 80.1 ppm (CH), and 58.7 ppm (CH_2_) confirmed the formation of CFCO.

### 3.4. Synthesis and Characterization of Cardanol-Based Non-Isocyanate Polyurethane

**Figure 10:**
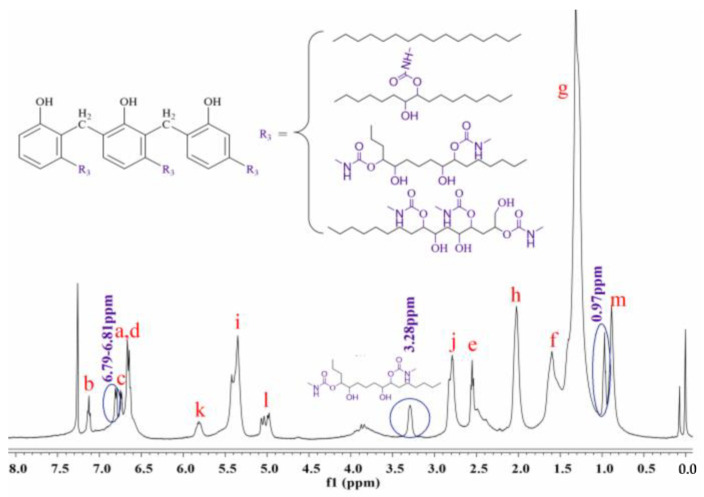
^1^H NMR spectra of cardanol-based non-isocyanate polyurethane (NIPU).

The ^1^H NMR spectrum of non-isocyanate polyurethane (NIPU) synthesized using cardanol as a starting material is shown in Figure 10. It was known that both the primary and secondary hydroxyl groups could be formed along the hydroxyurethane backbone due to the two different ring-opening pathways of the cyclic carbonates. Spectral peaks at 2.92–3.41 ppm and 6.79–6.81 ppm confirm the structure of the urethane group, possibly related to proton resonances on the urethane groups. The signal at 0.97 ppm was assigned to the methylene protons [37].

**Figure 11:**
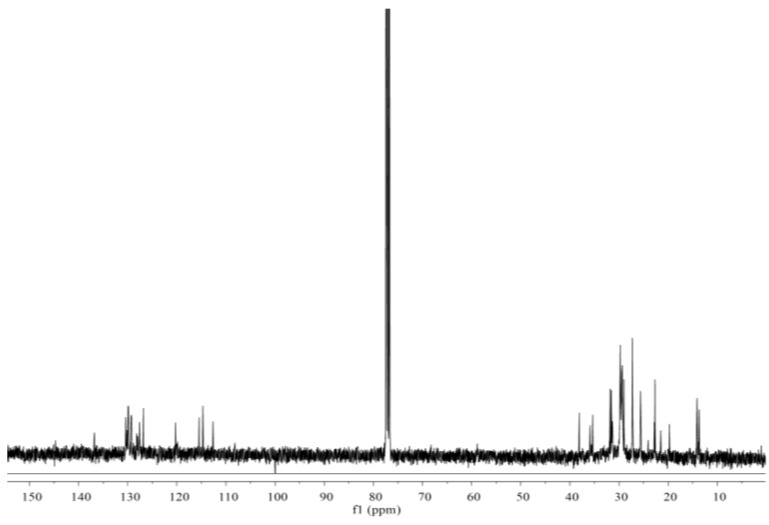
^13^C-NMR spectrum of cardanol-based non-isocyanate polyurethane (NIPU).

The chemical structures of the non-isocyanate polyurethane were further confirmed through ^13^C NMR analysis. Figure 11 is an example of the ^13^C NMR spectrum of typical NIPU synthesized from 1,6-hexanediamine. As the reaction time increased, the intensity of these two peaks (80.1 ppm (CH) and 58.7 ppm (CH_2_)) gradually decreased. After the reaction, these peaks almost disappeared, indicating the consumption of the cyclic carbonate [38,39].

**Figure 12:**
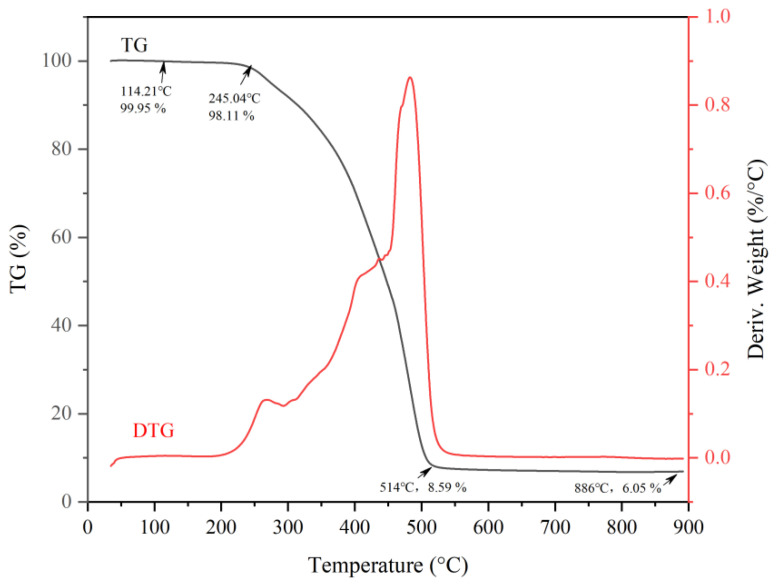
TG-DTA thermogram of cardanol-based non-isocyanate polyurethane (NIPU).

The thermal stability of the resin was analyzed using a thermogravimetric analyzer (TGA), as shown in Figure 12. The thermogram exhibits two degradation processes. The first degradation process occurs within the temperature range of 114.21 °C–245.04 °C, with a mass loss of 1.64%. This is attributed to the loss of water, impurities, unreacted cardanol monomers, and the breaking of fatty chain linkages in the aromatic ring of cardanol [31,38]. The second stage of degradation occurs around the temperature range of 245.04 °C to 534 °C. This is due to the depolymerization and further decomposition of the NIPU polyurethane structure. The exothermic peak observed in the DTA within the temperature range of 458 °C to 534 °C supports the decomposition of the polyurethane, which leads to a mass loss of 89.52% [40]. Taking into account the analysis data and other factors, we can conclude that cardanol-based polyurethane exhibits satisfactory thermal stability under high-temperature conditions. However, it is still important to pay attention to appropriate process conditions and environmental requirements to ensure its performance and durability when in use.

## 4. Conclusions

This paper focuses on the improvement and innovation of cardanol-based non-isocyanate polyurethane in terms of its unique structure and properties, including raw materials, equipment, and synthetic routes. Cardanol and formaldehyde were used as raw materials for synthesizing cardanol formaldehyde oligomer (CFO) through a citric acid-catalyzed reaction. The optimal reaction conditions were a molar ratio of cardanol to formaldehyde of 1:0.7, a reaction temperature of 120 °C, a reaction time of 5 h, and a catalyst amount of 1% (wt) cardanol, resulting in a brownish-red product. The cardanol formaldehyde oligomer was then further reacted with 3-chloroperbenzoic acid to oxidize the double bonds in the side chain, forming epoxy compounds. A more environmentally friendly method for replacing m-chloroperoxybenzoic acid with hydrogen peroxide has also attracted attention in this work. Here, epoxy compounds were reacted with CO_2_ to produce viscous cyclic carbonates, which required heating before the subsequent reaction. The feasibility of using a monomeric cardanol formaldehyde cyclocarbonate oligomer to react with 1,6-hexanediamine for the development of cardanol-based non-isocyanate polyurethane (NIPU) via a nonisocyanate pathway was also explored in this study. The synthesis of cardanol-based non-isocyanate polyurethane, obtained by reacting cyclic carbonates with amines, exhibited a stable thermal performance within the tested temperature range, as assessed using thermogravimetric analysis (TGA). The thermogravimetric curve showed significant thermal decomposition or degradation of the cardanol-based non-isocyanate polyurethane in the temperature range of 245.04 °C to 534 °C. The onset temperature of mass loss was 245.04 °C, indicating that approximately 89.52% of the mass loss was due to the thermal decomposition of cardanol-based non-isocyanate polyurethane occurring below this temperature.

In general, this study provided a novel route for the synthesis of bio-based NIPU from renewable resources. It would be of great interest to investigate the effects of catalysts on the polyaddition reaction between cyclic carbonate and a variety of diamines (e.g., cyclic, aromatic) for the synthesis of other NIPU in future studies, and in the future, we aim to make non isocyanate polyurethane coating and foam for field applications.

## Figures and Tables

**Table 1:** Molecular weight and polydispersity index of raw cardanol and cardanol formaldehyde oligomer.

Peak No	Mp g/mol	Mn g/mol	Mw g/mol	Mz g/mol	M_z+1_ g/mol	Mv g/mol	PD g/mol
1	5237	5131	6360	7857	9433	6155	1.24
2	384	354	466	614	775	447	1.32

## Data Availability

The data presented in this study are available from the corresponding author upon request.
